# Vaccine-Derived Polioviruses Not Detected by Global Surveillance Screening Assay

**DOI:** 10.3201/eid2110.150702

**Published:** 2015-10

**Authors:** Deepa K. Sharma, Uma P. Nalavade, Swapnil Y. Varose, Jagadish M. Deshpande

**Affiliations:** Enterovirus Research Centre, Mumbai, India

**Keywords:** Vaccine derived poliovirus, Real-Time RT-PCR, Poliomyelitis, AFP surveillance, polio eradication, viruses

**To the Editor:** The Global Polio Laboratory Network (GPLN) and the World Health Organization’s Polio Eradication Initiative (GPEI) accord high priority to detecting all vaccine-derived polioviruses (VDPVs) because they are neurovirulent and have the potential to cause outbreaks of poliomyelitis and establish poliovirus circulation. In patients with immunodeficiency diseases, persistent infections may become established with live oral poliovirus vaccine (OPV) and develop into VDPVs (*1*). Laboratories of the GPLN use standard procedures for virus isolation, identification, and intratypic differentiation (*2*). The real-time reverse transcription PCR (rRT-PCR) VDPV screening assay became available to the GPLN in 2009. Poliovirus isolates that do not become amplified in the VDPV assay are subjected to complete sequencing of viral protein (VP) 1. VDPVs are isolates of Sabin OPV origin that have incorporated >6 nucleotide substitutions (Sabin2) or ≥10 nucleotide substitutions (Sabin1 and Sabin3) in the VP1 region. The VDPV assays were found to be 100% specific for all 3 poliovirus types, 100% sensitive for Sabin1 and Sabin3, and 76% sensitive for Sabin2 (*3*). Among all cases of circulating VDPV infection reported globally from 2000 to 2013, 10.95%, 97.1%, and 1.8% of cases were caused by types 1, 2, and 3, respectively (*4*). 

To mitigate the risk for infection with VDPV type 2, GPEI envisions a simultaneous global switch from trivalent OPV to bivalent OPV (Sabin1 and Sabin3)—that is, withdrawal of Sabin2—beginning in April 2016 (*5*). Any type 2 polioviruss detected thereafter will need characterization. Here we report VDPV isolates that escaped detection by the VDPV screening assay used in the GPLN.

Type 3 VDPV was identified in a 1.2-year-old girl with onset of acute flaccid paralysis (AFP) on January 2008. Sabin3 was isolated from stool sample 1 (R46064), collected 11 days after onset of paralysis. Stool sample 2, collected on the 13th day after paralysis, was negative for virus. R46064 was reported as “Sabin-like” by the intratypic differentiation tests (ELISA and conventional RT-PCR) used in the GPLN in 2008 (*6*,*7*). The patient had residual weakness compatible with paralytic poliomyelitis; therefore, the isolate was characterized in detail. Complete genome sequence of R46064 showed that the major attenuation sites reverted to wild type at nt 472 (U→C) in the 5′ untranslated region (UTR) and nt 2034 in VP3 (U→C). The capsid region contained 18 nt substitutions, of which 12 were in the VP1 region (Technical Appendix Table). Seven amino acid changes occurred, including 2 at the antigenic site NAg1 (Technical Appendix Figure 1). The isolate was a recombinant with species C enterovirus in the noncapsid region. R46064 was a VDPV3 isolate by definition. Investigations showed that VDPV3 was not a part of any outbreak.

R46064 produced Sabin3-like results in the VDPV screening assay. R46064 gave false-negative test results because the isolate had a Sabin3 vaccine sequence in the regions corresponding to the probe and primers of the VDPV assay (Technical Appendix Figure 2).

Type 2 VDPV was found in an immunocompetent girl, 3.5 years of age, in March 2014. Sabin2 was isolated from 2 stool samples collected 5 and 8 days after onset of AFP. Sabin2, isolated from stool sample 1 (R93150), was amplified in the VDPV screening assay (reported as Sabin2-like); the isolate from stool sample 2 (R93152) failed to become amplified (reported for sequencing).

VP1 sequencing of R93152 showed 6 nt substitutions; therefore, it was reported as VDPV2. R93150 was also sequenced to find out whether it contained the Sabin2 homotypic mixture. VP1 sequence of R93150 showed 6 nt substitutions and no evidence of mixed bases. Substitution was not found at VP1 nt 427/428, the main target of the VDPV screening assay.

Complete genome sequence analysis revealed that both isolates contained reversion of the major attenuating site at nt 481 in the 5′ UTR. The genomes of both isolates showed no recombination. Both isolates showed 15 nt substitutions in the capsid region, when compared with Sabin2 (Technical Appendix Table); only 5 substitutions were common to both isolates. R93150 had 4 aa changes; 1 change was at the antigenic site NAg2. R93152 showed 6 aa changes; 2 changes were at NAg3a and NAg3b (Technical Appendix Figure 1). Thus, VDPVs of 2 distinct VDPV2 lineages were excreted by the patient; 1 isolate was identified as VDPV2, but the other was missed. Although the patient was identified as having VDPV2 infection by the current algorithm, if special interest had not been taken to characterize both isolates, we would not have detected the VDPV2 that produced false-negative results in the screening assay.

The occurrence of VDPV2 and VDPV3 as described above may be rare. However, GPLN laboratories are unlikely to detect VDPV strains that produce false-negative results in the VDPV screening assay. False negatives are of greatest concern to the GPEI because they could impede timely detection of VDPV infections (*3*,*8*). Our results point out the need for reporting and inventorying VDPVs that give a false-negative reaction in the screening assay. This action would help clarify how to further refine the screening assays.

Technical Appendix Nucleotide substitutions in the 5′UTR and capsid region of type 2 and type 3 vaccine-derived polioviruses (VDPV), amino acid changes at neutralizing antigenic sites, and nucleotide sequence alignment of VDPV2 and VDPV3 with corresponding Sabin vaccine virus sequences.
