# Supplementary material for: Vaccine-Derived Polioviruses Not Detected by Global Surveillance Screening Assay
Source: Emerg Infect Dis. 2015 Oct;21(10):1880–1. doi: 10.3201/eid2110.150702 (PMC4593449; doi:10.3201/eid2110.150702)
Supplement: Technical Appendix — Nucleotide substitutions in the 5′UTR and capsid region of type 2 and type 3 vaccine-derived polioviruses (VDPV), amino acid changes at neutralizing antigenic sites, and nucleotide sequence alignment of VDPV2 and VDPV3 with corresponding Sabin vaccine virus sequences. [file 15-0702-Techapp-s1.pdf]

Article DOI: http://dx.doi.org/10.3201/eid2110.150702

Vaccine-Derived Polioviruses Not Detected by Global Surveillance Screening Assay

Technical Appendix

Technical Appendix Table. Nucleotide substitutions in 5′ UTR and capsid region of type 2 and type 3 VDPV strains*

	Type 2 VDPV			Type 3 VDPV	
Region	Nucleotide no.	R93150	R93152	Nucleotide no.	R46064
5′UTR	481	A—>G	A—>G	472	T—>C
	681		T—>C		
	707	A—>T			
	730		T—>C		
VP4	831	T—>C		844	A—>G
	927		C—>A		
VP2	1059	C—>T	C—>T	1066	T—>C
	1164	T—>G		1567	T—>C
	1168		**C—>T**		
	1469	**A—>G**			
	1507	C—>T			
	1704	G—>A			
VP3	1857		C—>T	2034	**T—>C**
	1943		**G—>T**	2278	T—>C
	2025		G—>A	2401	T—>C
	2046	C—>T			
	2055	A—>G	A—>G		
	2420	**A—>G**	**A—>G**		
	2475		**G—>A**		
VP1	2490	T—>C	T—>C	2546	**A—>G**
	2525	**A—>G**		2551	A—>G
	2739	C—>T		2636	**G—>A**
	2766		T—>C	2760	**C—>M**
	2829	T—>C		2766	**C—>T**
	2908		**A—>G**	2917	T—>C
	2958	T—>C		2967	**C—>T**
	2992	**A—>G**	**A—>G**	2972	**A—>G**
	3057		A—>G	2973	A—>G
	3381		T—>G	3067	G—>A
				3226	T—>C
				3316	A—>G

*Nucleotide substitutions are shown as Sabin—->Isolate; Nucleotide substitutions causing amino acid changes are shown in bold type; The complete genome sequences of R93150, R93152, and R46064 were submitted to GenBank (accession nos. KR259356–KR259358). UTR, untranslated region; VDPV, vaccine-derived poliovirus.

	VP2			VP3		VP1		
	NAg3b	NAg2	NAg2	NAg3a	NAg3b	NAg1	NAg2	NAg3a
	71	164	268	54	75	88	222	287
Sabin 2	WRK	TNATNPARNPRT	PRTQ	PLNLTSQRVELSD	TAHSDT	AIIEVDNDAPTKRASRLFS	STEGD	KDGLT
R93150	...	........**S**...	....	....	......	....	.....	.....
R93152	.**C**.	....	....	.....**I**.......	......	....	.....	.....
Sabin 3	WGK	NAVTSPKREPKF	PKFQ	PLNLESTKVTLSD	SADLSQ	IEVDNEQPTTRAQKLFAMW	QIGDS	NNLDP
R46064	...	....	....	....	......	.......**Q**.**I**.........	.....	.....

Technical Appendix Figure 1. Amino acid changes at neutralizing antigenic sites (NAg) of VDPVs. The number indicates the first amino acid position within the antigenic site.

Sabin2	AAACTGGAATTTTTCACATATTCGAGATTT**GACATGGAGTTCACTTTTG**TGGTCACCTCA
R-93150	AAACTGGAATTTTTCACATATTCGAGATTTGACATGGAGTTCACTTTTGTGGTCACCTCA
R-93152	AAACTGGAATTTTTCACATATTCGAGATTTGACATGGAGTTCACTTTTGTGGTCACCTCA
	**
Sabin2	AACTA**CATTGATGCAAATAAC**GGACATGCATTGAACCAAGTTTATCAGATAAT**GTATATA**
R-93150	AACTACATTGATGCAAATAACGGACATGCATTGAACCAAGTTTATCAGATAATGTACATA
R-93152	AACTACGTTGATGCAAATAACGGACATGCATTGAACCAAGTTTATCAGATAATGTATATA
	****** *** ***
Sabin2	**CCACCCGGAG**CACCTATCCCTGGTAAATGGAATGACTATACGTGGCAGACGTCCTCTAAC
R-93150	CCACCCGGAGCACCTATCCCTGGTAAATGGGATGACTATACGTGGCAGACGTCCTCTAAC
R-93152	CCACCCGGAGCACCTATCCCTGGTAAATGGGATGACTATACGTGGCAGACGTCCTCTAAC
	****************************** *****************************
Sabin3	GTTCGTGTTGTCAATGATCACAACCCCACTAAAGTAACCTCCAAAGTCCG**CATTTACATG**
R-46064	GTTCGTGTTGTCAATGATCACAACCCCACCAAAGTAACCTCCAAAGTCCGCATTTACATG
	***************************** ******************************
Sabin3	**AAACCCAAAC**ACGTACGTGTCTGGTGCCCTAGACCGCCGCGCGCGGTACCTTATTATGGA
R-46064	AAACCCAAACACGTACGTGTCTGGTGCCCTAGACCGCCGCGCGCGGTACCTTATTATGGG
	***
Sabin3	CCAGGGGTGGACTA**TAGGAACAACTTGGAC**CCCTTA**TCTGAGAAAGGTTTGACCA**CATAT
R-46064	CCAGGGGTGGACTATAGGAACAACTTGGACCCCTTATCTGAGAAAGGTTTGACCACATAT
	**

Technical Appendix Figure 2. Nucleotide sequence alignment of VDPV2 and VDPV3 with corresponding Sabin vaccine virus sequences. Primer and probe regions are shown in bold type.
